# Evaluation of psychopharmacological and neurosafety profile of Swas Kas Chintamani Ras (SKC) in Swiss-Webster mice 

**Published:** 2018

**Authors:** Tarequl Islam, Md. Saddam Hussain, Mohammad Nurul Amin, Ashraful Mahmud Tuhin

**Affiliations:** 1 *Department of microbiology, Noakhali Science and Technology University, Sonapur-3814, Noakhali, Bangladesh*; 2 *Department of pharmacy, Noakhali Science and Technology University, Sonapur, Noakhali-3814, Bangladesh*; 3 *Department of pharmacy, Atish Dipankar University of Science and Technology, Banani, Dhaka-1213, Bangladesh*

**Keywords:** SKC, Psychopharmacological, Neurosafety, Number of rearing

## Abstract

**Objectives:**

Swas Kas Chintamani Ras (SKC) is an ayurvedic preparation indicated for respiratory diseases. Our study was aimed to determine the psychopharmacological and neurosafety profile of SKC.

**Materials and Methods:**

Psychopharmacological effects and neurosafety profile of this drug were determined by nine complementary test methods namely, open field, locomotor activity, hole cross, hole board test, elevated plus maze, staircase, forced swimming test, and rotarod test. Male mice (Swiss-Webster strain, 20-40 g body weight) bred in the Animal House of the Department of Pharmacy, Jahangirnagar University, were used for the pharmacological experiments.

**Results:**

The drug decreased total ambulation and movement in the central region and standing up behavior and lowered emotional defecation. The drug also made the mice to take a shorter time to come out of the cage. Also, animals spent less time in open arm and the movement in the closed arm and locomotors reduced (p=0.003), where a number of rearing (p=0.04) behaviour indicating possible anxiolytic activity. Also, no signs of anti-depressant activity were observed among SKC-treated group.

**Conclusion:**

We concluded that our drug showed no neurotoxic effect and it also showed some beneficial neuropharmacological properties.

## Introduction

Ayurvedic system of medicine is the most ancient medicine system originated in India about 3000 years ago (Ramakrishna et al., 2006 ▶). Most of the primitive traditional methods of healing such as Tibetan, Chinese and Greek medicine have been influenced by Ayurvedic medicine (Mandell et al., 2014 ▶; Dash, 1984 ▶; Dastur, 1960 ▶; Mishra and Chandra, 2010 ▶). In Indian sub-continent, nearly 80% of the population is reported to use Ayurveda and medicinal plants to help meet their primary health care needs. Ayurveda can help maintain health in a person by maintaining the individual body‘s mind and spirit in perfect equilibrium with nature (Nadkarni, 1976 ▶; Verma, 1991 ▶; Sadhana et al., 2012). A well-known herbal medicine is Swas Kas Chintamani Ras, which is available as tablet formulation and used for treatment of heart diseases, lung diseases, diabetes, cough, cold and other respiratory diseases. It helps to improve strength and immunity (Mishra and Chandra, 2010 ▶; Nadkarni, 1976 ▶). This herbal preparation contains heavy metal ingredients, due to which it is recommended to only be taken under strict medical supervision (Verma, 1991 ▶; Sadhana et al., 2012). The tablets are normally 125 – 250 mg and taken once or twice a day, before or after meal or taken as directed by an Ayurvedic practitioner. It is also advised to be used along with long pepper and honey and is administered for a period of one month normally. This medicine has traditionally been administered along with a water decoction of wheat. Many companies promote this product as Chintamani Ras with GOLD. This product contains purified and processed mercury, purified and processed sulphur, pPurified and processed silica, iron bhasma, tin bhasma, purified asphaltum, gold bhasma, silver bhasma, leadwort, *Eclipta alba* and *Terminalia arjuna*. If gold bhasma is not included, it can not be called Chintamani Ras (Hebbar BAMS, 2015 ▶). Thus, self-medication of this medicine may be dangerous since it contains heavy metal ingredients. That is why patients are advised to take this medicine at corrected doses, for a limited duration of time and under close supervision of a doctor. Over-dosage may cause severe poisonous effects. It should not be prescribed during pregnancy and lactation and for children (Hebbar BAMS, 2015 ▶). 

Studies on behavioral patterns are carried out to get a clear picture of the effect of the drugs by investigation of the pattern of behavior and emotional defecation of the animals (Boissier and Simon, 1964 ▶). Ayurvedic medicine has a good safety profile (Ernst, 2002 ▶). But, a recent study has reported that heavy metal content of the Ayurvedic preparations (e.g. lead) exhibits numerous toxicity (Keen et al., 1994 ▶). The safety profile of most of the Ayurvedic medicine preparations has not yet been completely investigated though studied drugs contain heavy metals requiring research to be carried out in this regard. As Ayurved pharma is becoming available in the international market with the goal of reaching herbal access for each and every part of world, elucidation of safety profile of Ayurvadic drugs is needed validate their use. After reviewing the current literature, we found that no research has been executed to validate claims of Swas Kas Chintamani Ras (SKC) as a whole aggregate for psychopharmacological activities. Hence, the present study examines psychopharmacological and neurosafety profile of SKC in Swiss-Webster mice, which was done as per the recommended approach in Ayurveda. If found to be effective, SKC may be considered a beneficial therapeutic adjuvant or for a candidate for prevention of psychopharmacological disorder. 

## Materials and Methods

**Collection of the ayurvedic formulation**

For evaluating psychopharmacological and neurosafety profile of SKC, it was collected from Sree Kundeswari Aushadhalaya Ltd, Chittagong, Bangladesh. 

**Experimental animal**

Male mice (Swiss-Webster strain, 20-40 g body weight) bred in the Animal House of the Department of Pharmacy, Jahangirnagar University, were used for the pharmacological experiments. They were kept in cages (30 × 20 × 13 cm) and soft wood shavings were employed as bedding in the cage. Animals had free access to standard laboratory food and tap water ‘*ad libitum’* and were maintained under the natural day-night cycle. They were fed with “mouse chow” (prepared according to the formula developed at BCSIR, Dhaka). Before starting an experiment, the animals were carefully marked on different parts of their body, which was later used as an identification mark for a particular animal so that the response of a particular mouse prior to and after the administration could be noted separately. 

**Doses Used In Different Experiments**

For Open Field test 100, 200 and 400mg/kg body weight (BW), for locomotor test 100, 200 and 400mg/kg BW, for hole cross test 100, 200 and 400mg/kg BW, for hole board test 100, 200 and 400 mg/kg BW, for elevated plus maze test 100, 200 and 400mg/kg BW, for staircase test 100mg /kg BW, for forced swim test 100mg/kg BW, and for rotarod test 100,200 and 400 mg/kg BW were used.

**Psychopharmacological activity test**

**The open field test**

In this experiment, the method developed by Gupta (1971) ▶ was employed (Gupta et al., 1971 ▶). The floor of an open field of half square meter was divided into a series of squares, each alternately colored in black and white. The apparatus had a wall of 40 cm. The number of squares, traveled by the animal, was recorded for a period of two minutes. All studies were carried out between 8 a.m. and 5 p.m.

**Locomotor activity in mice**

The Ugo Basile model no. 47420 Activity Cage, is great value to record spontaneous co-ordinate activity of mice (in groups of two) and measure variations in this activity with respect to time. The 47420 multiple activity cage package comprises an electronic unit 7441 and an I.R. Beam Cage, which consists of an animal cage of clear Perspex, 40×40cm, designed with two sets of emitter/sensor arrays for horizontal and vertical activity. This set-up can accept up to 5 additional cages, for a total of 6. The Electronic Units incorporate a graphic display, a thermal printer and a serial port RS232 for direct connection to the PC using the software Cat. 52050. The graphic display presents all available commands. The operator sets the experiment configuration via the keyboard located below the display. The activity data are displayed at pre-set intervals and printed/routed to the computer according to the selected configuration. The data can be customized by adding animal & experiment numbers, gender, etc. Also, 7441 is provided with an internal memory, capable of storing the data of several experiments, to be unloaded to the PC later. All studies were carried out between 8 a.m. and 5 p.m.

**Hole cross test**

In this experiment, the method of Takagi et al (1971) was employed (Takagi et al., 1971 ▶). In a (30 × 20 × 14 cm), a hole of 3 cm in diameter at a height of 4.5 cm from the floor, was made on the dividing wall. Spontaneous movement of the animals through the hole from one chamber to the other was counted for a period of two hours. The observation was conducted 30, 60, 120 and 240 min after oral administration of test drugs and was compared with control animal administered with normal saline. All studies were carried out between 8 a.m. and 5 p.m.

**Hole board test**

The Hole Board test has been conceived to study the behavior of the mouse confronted with a new environment (head plunging stereotype) according to the method devised by Boissier, Simon and Lwoff (Boissier and Simon, 1964 ▶). This experiment was carried out using the following method of Nakama et al, 1972 ▶ (Nakama et al., 1972 ▶). A total of 16 holes, each 3 cm in diameter, were presented to each mouse in a flat space of 25 square centimeters. Each of the animals was transferred carefully to one corner of the field and the number of ambulation (expressed as the number of holes passed), head dipping and number of fecal boluses excretion was recorded for a period of 2 minutes at 30 minutes before as well as 30, 60, 120 and 240 minutes after the treatment and compared with the control animals administered with distilled water (Nakama et al., 1972 ▶). All studies were carried out between 8 a.m. and 5 p.m.

**Elevated plus maze test**

The elevated plus-maze, a modification of the method used by Lister (1987) (Lister, 1987), consisted of two open arms (30 × 5 × 0.5 cm) and two closed arms (30 × 5 × 15 cm) with an open roof, arranged in a way that two pairs of identical arms were opposite to each other. Arms emerged from a central platform (5 × 5 cm), and the entire apparatus was raised to a height of 50 cm above the floor level. The maze was constructed from black plexiglass. Mice were administered with test compound and placed individually in the center of the maze, facing one of the open arms. The number of entries into both open and enclosed arms and the amount of time spent in the open arms was recorded. Each test lasted for 5 min and each mouse was tested only once. The apparatus was cleaned between each test. The test compounds were administered 30 min before the test at a volume of 10 ml/kg body weight. All tests were conducted between 08:00 and 14:00.

**Staircase test**

The apparatus consisted of a white PVC enclosure with a five-step staircase. The box is placed in a room with constant lighting, isolated from external noise, and thermostatically controlled. Native male mice weighing 21± 3 g were used in these studies. The day before the test, the animals were randomly divided into groups of 12 mice in plastic cages. All animals used for a single experiment were placed at the same height in the animal house. They were transferred to the laboratory at least 1 hr before the start of the test. Each animal was used only once. The animal was placed singly on the floor of the box with its back to the staircase. The number of steps climbed and the number of rears were counted during a 3-min period. A step was considered ”climbed” only if the mouse had placed all four paws on the step. The number of steps descended was not taken into account in order to simplify the observations. After each test, the box was rapidly cleaned to eliminate any olfactory cue which might affect the next animal's behavior. Experimental drugs were administered orally (10 mg/kg) 60 min before the test to groups of 12 mice. In each experiment, a control group received only distilled water. The treatments were randomized, and the observer was unaware of the treatment given to each group (blind method) (Simiand et al., 1984). All studies were carried out between 8 a.m. and 5 p.m. 

**Forced swim test**

The most widely utilized animal model of antidepressant action is the forced swim test (FST). The traditional version of this test was developed by Roger Porsolt and colleagues (Porsolt et al., 1977) and comprises exposing mice to a 15-min swim 24 h before a 5-min test exposure in 15–18 cm of 25^o^C water. Following an initial period in which the rat produces escape-directed behaviors, it will adopt an immobile posture, which is believed to reflect either a failure to show a persistent escape-directed behavior or a passive behavior to cease active forms of coping with the stressful stimuli. A wide range of clinically effective antidepressants has been shown to increase the time that the rat spends in active escape behaviors. All studies were carried out between 8 a.m. and 5 p.m.

**Rotarod tests with constant speed model**

The “Rota-Rod” technique has been originated by a 1957 paper of Dunham and Miya and is of great value in research screening drugs which are potentially active on motor co-ordination. Motor performance was evaluated by using a rotarod apparatus (Techno, Lucknow, India) (Nakama et al., 1972 ▶). All studies were carried out between 8 a.m. and 5 p.m.

**Statistical analysis**

Data were presented as Mean ± SEM (Standard Error of the Mean). Unpaired "t" tests were done for statistical evaluations. SPSS (Statistical Package for Social Science) for windows (Ver. 11) was applied for the analysis of data. A p<0.05 was taken as significance.

## Results

As far none of the data were found to statistically significant (p>0.05) but drug SKC represent some permissible results in total and center ambulation in open field test and the result is tabulated in Tables 1 and 2. All three doses of SKC (100, 200 and 400 mg/kg) exerted overall decreases in total ambulation, while noticeable exceptions was found at min 30 where all doses (100, 200 and 400 mg/kg) showed increases in total ambulation. Moreover, all these data were found statistically non-significant compared to ??? (p>0.05). Male mice treated with SKC showed an overall decrease in total movement in the center region (100, 200 and 400 mg/kg) and data were comparable to the results of the respective control group. Again, the standing up behavior in the SKC-treated male mice was decreased at all three experimental doses throughout the experimental period (Table 3); But, when data was compared with results of the control group none of the differences were found statistically significant (p>0.05). Finally, at all three doses (100, 200, and 400mg/kg), SKC showed a gradual increase in emotional defecation in open field test at 240 min and differences were found to be statistically non-significant (p>0.05) when compared to the corresponding control group (Table 4).

**Table 1:** The effect of SKC (100, 200 and 400 mg/kg) on total ambulation in the open field test.

Conc.	Group	Ctrl (n=6)	SKC (n=6)	95% confidence interval
100 (mg/Kg)	Min 0	82.50±9.54	88.00±11.19	­38.270 to 27.270
	Min 30	54.00±18.03	64.50±16.52	­64.946 to 43.946
	Min 60	33.66±9.51	22.33±6.38	­14.184 to 36.850
	Min 120	37.50±13.01	19.83±14.14	­35.208 to 50.541
	Min 180	29.83±14.14	12.83±5.07	­16.460 to 50.460
	Min 240	21.17±6.25	16.00±7.13	-15.958 to 26.292
200 (mg/Kg)	Min 0	20.67±6.69	34.50±15.69	­51.853 to 24.186
	Min 30	9.33±1.81	25.83±12.07	­47.466 to 14.466
	Min 60	19.67±13.04	12.17±9.23	­28.113 to 43.113
	Min 120	20.00±8.69	8.17±4.08	­10.806 to 34.472
	Min 180	11.00±6.34	16.33±9.04	­29.948 to 19.281
	Min 240	13.17±6.05	7.17±3.18	­9.941 to 21.941
400 (mg/Kg)	Min 0	16.33±7.77	22.00±11.72	­37.001 to 25.668
	Min 30	4.67±3.46	8.50±7.02	­21.277 to 13.610
	Min 60	1.67±1.67	6.33±2.88	­12.086 to 2.753
	Min 120	9.17±4.53	20.00±8.03	­31.387 to 9.720
	Min 180	18.17±7.71	10.83±5.37	­13.614 to 28.281
	Min 240	20.67±11.68	4.83±2.47	­10.762 to 42.428

F.N: NS= Not Significant.

**Table 2.** The effect of SKC (100, 200 and 400 mg/kg) on the center ambulation in the open field test

Conc.	Group	Ctrl (n=6)	SKC (n=6)	95% confidence interval
100 (mg/Kg)	Min 0	2.67±1.08	0.17±0.17	­0.284 to 5.284
	Min 30	0.33±0.33	0.50±0.50	­1.505 to 1.172
	Min 60	0.17±0.17	0.17±0.17	­0.525 to 0.525
	Min 120	0.17±0.17	0.50±0.34	­1.180 to 0.513
	Min 180	0.17±0.17	0.00±0.00	­0.261 to 0.595
	Min 240	0.00±0.00	0.17±0.17	­0.595 to 0.261
200 (mg/Kg)	Min 0	0.00±0.00	0.00±0.00	0.000 to 0.000
	Min 30	0.00±0.00	0.33±0.33	­1.190 to 0.523
	Min 60	0.33±0.33	0.00±0.00	­0.523 to 1.190
	Min 120	0.00±0.00	0.00±0.00	0.000 to 0.000
	Min 180	0.33±0.33	0.33±0.33	­1.050 to 1.050
	Min 240	0.33±0.33	0.00±0.00	­0.523 to 1.190
400 (mg/Kg)	Min 0	0.00±0.00	0.00±0.00	0.000 to 0.000
	Min 30	0.00±0.00	0.00±0.00	0.000 to 0.000
	Min 60	0.17±.017	0.00±0.00	­0.261 to 0.595
	Min 120	0.17±.017	0.17±.017	­0.525 to 0.525
	Min 180	0.17±.017	0.17±.017	­0.525 to 0.525
	Min 240	0.17±.017	0.00±0.00	­0.261 to 0.595

N.B: NS= Not Significant.

**Table 3:** The effect of SKC (100, 200 and 400 mg/kg) on the standing up behavior in the open field test

Conc.	Group	Ctrl (n=6)	SKC (n=6)	95% confidence interval
100 (mg/Kg)	Min 0	9.33±2.73	8.00±1.98	­6.182 to 8.849
	Min 30	6.50±3.38	6.67±1.93	­8.842 to 8.509
	Min 60	4.83±1.14	2.17±1.60	­1.708 to 7.041
	Min 120	3.50±2.58	1.67±0.99	­4.320 to 7.987
	Min 180	0.83±0.65	0.33±0.33	­1.135 to 2.135
	Min 240	1.17±0.60	0.83±0.65	­1.645 to 2.312
200 (mg/Kg)	Min 0	0.33±0.33	0.67±0.67	­1.994 to 1.327
	Min 30	0.33±0.21	0.50±0.34	­1.061 to 0.727
	Min 60	1.50±1.50	0.33±0.33	­2.257 to 4.590
	Min 120	0.83±0.65	0.33±0.21	­1.031 to 2.631
	Min 180	0.50±0.34	1.00±0.68	­2.201 to 1.201
	Min 240	0.67±0.42	0.00±0.00	­0.417 to 1.750
400 (mg/Kg)	Min 0	0.33±0.33	1.00±0.52	­2.036 to 0.702
	Min 30	0.50±0.50	0.17±0.17	­0.841 to 1.507
	Min 60	0.17±0.17	0.00±0.00	­0.204 to 0.538
	Min 120	0.50±0.34	0.17±0.17	­0.513 to 1.180
	Min 180	1.50±0.81	0.33±0.33	­0.917 to 3.251
	Min 240	0.67±0.67	0.00±0.00	­1.047 to 2.380

N.B: NS= Not Significant.

**Table 4:** The effect of SKC (100, 200 and 400 mg/kg) on the emotional defecation in the open field test.

Conc.	Group	Ctrl (n=6)	SKC (n=6)	95% confidence interval
100 (mg/Kg)	Min 0	0.17±0.17	0.17±0.17	­0.52 to 0.525
	Min 30	0.33±0.21	0.33±0.21	­0.664 to 0.664
	Min 60	0.50±0.34	0.17±0.17	­0.513 to 1.180
	Min 120	0.00±0.00	0.00±0.00	0.000 to 0.000
	Min 180	0.17±0.17	0.33±0.33	­0.997 to 0.663
	Min 240	0.00±0.00	0.50±0.34	­1.378 to 0.378
200 (mg/Kg)	Min 0	1.33±0.42	2.00±0.58	­2.259 to 0.926
	Min 30	1.33±0.61	1.50±0.72	­2.279 to 1.940
	Min 60	1.17±0.48	1.50±0.56	­1.977 to 1.310
	Min 120	1.33±0.49	1.67±0.67	­2.182 to 1.516
	Min 180	0.00±0.00	0.33±0.33	­1.676 to 0.409
	Min 240	1.12±0.54	0.33±0.21	­0.566 to 2.232
400 (mg/Kg)	Min 0	0.17±0.17	0.50±0.34	­1.180 to 0.513
	Min 30	0.00±0.00	0.00±0.00	0.000 to 0.000
	Min 60	0.00±0.00	0.33±0.33	­1.190 to 0.523
	Min 120	0.17±0.17	0.17±0.17	­0.525 to 0.525
	Min 180	1.00±0.45	0.67±0.33	­0.909 to 1.576
	Min 240	0.33±0.33	0.33±0.21	­0.878 to 0.878

N.B: NS= Not Significant

SKC-treated mice exerted locomotor activity at the 4^th^ and 5^th ^hour at the dose of 100 mg/kg, at the 3^rd^, 4^th^ and 5^th^ hour at the dose of 200 mg/kg and at the dose of 400 mg/kg SKC showed locomotor activity from the 1^st^ hr to end of the 5th hour (Table. 5). All three doses of SKC exerted a decreased at the 6^th^ hour which was significantly different from that of the control group (p<0.05). Again 200 and 400 mg/kg of SKC showed significant increases in locomotor activity (p<0.05) at 195 and 60 min, respectively. Mice treated with SKC 100, 200 and 400 mg/kg showed increases in the motor activity with no domino effect when study carried out at hole cross board (Table 6). But, all of the results were non-significant different from the control group (p>0.05). An interesting significant (p<0.05) difference was found between SKC 100mg/kg and the control group where total ambulation in the hole board test was decreased at the 1st hr of the experimental period. Overall decreases in head dipping activity were observed at all three doses. However, all of the results were found statistically insignificant (p>0.05) while compared with control group. SKC-treated group showed a significant (p<0.01) increase in emotional defecation at 200 mg/kg from 30 min to 120 min (Table 7).

**Table 5:** The effect of SKC (100, 200 and 400 mg/kg) at the 1^st^ to the 6^th^ hr of the locomotor test

Conc.	Group	Ctrl (n=6)	SKC (n=6)	95% confidence interval
100 (mg/Kg)	Hr1	22.92±3.51	21.93±3.85	-5.259 to 11.255
	Hr2	2.94±1.08	5.54±2.93	-9.558 to 4.372
	Hr3	6.28±2.27	3.66±2.18*	-4.395 to 9.625
	Hr4	11.76±2.04	14.44±1.25	-7.993 to 2.647
	Hr5	9.03±1.97	9.53±1.82	-6.483 to 6.48
	Hr6	5.18±1.93	4.53±0.78	-3.972 to 5.286
200 (mg/Kg)	Hr1	40.78±3.76	38.92±3.09	-8.976 to 12.686
	Hr2	5.89±1.29	5.41±1.15	-3.834 to 4.808
	Hr3	4.94±1.36	5.72±1.31*	-4.988 to 3.425
	Hr4	2.92±1.33	5.69±2.19	-8.500 to 2.950
	Hr5	1.71±0.72	3.08±1.42	-4.919 to 2.166
	Hr6	2.45±0.98	2.25±2.28*	-3.394 to 3.797
400 (mg/Kg)	Hr1	35.17±13.39	36.43±6.80*	-34.742 to 32.215
	Hr2	10.49±1.02	12.89±1.56	-6.563 to 1.753
	Hr3	5.61±1.25	6.62±0.83	-4.348 to 2.338
	Hr4	2.62±0.92	2.77±1.23	-3.589 to 3.283
	Hr5	2.62±0.92	3.92±0.87*	-4.127 to 1.527
	Hr6	2.65±1.08	1.65±0.35	-1.789 to 3.783

N.B: Here, Hr stands for hour,

* indicates p<0.05.

All three doses non-significantly (p>0.05) decreased emotional defecation and overall increased effect in emotional defecation throughout the experimental period in comparison to the control group (Tables 8 and 9). At 200 mg/kg, SKC showed an increase in the time spent in the open arm from 120 min to the end of 240 min. At 400 mg/kg, SKC-treated male mice did not exhibit any interesting results rather decreasing effect (Table 10). SKC at all three doses (100, 200, and 400 mg/kg) exerted overall decrease in total movement in close arm on elevated plus maze test in comparison to the respective control group. At the dose 100mg/Kg, exceptions were being observed at 180 min and 240 min. But all of the results were found statistically insignificant (p>0.05) while compared with control group (Tables 11).

**Table 6:** The effect of SKC (100, 200 and 400 mg/kg) in the hole cross test.

Conc.	Group	Ctrl (n=6)	SKC (n=6)	95% confidence interval
100 (mg/Kg)	Min 0	0.00±0.00	0.16±0.16	-0.595 to 0.261
	Min 30	2.00±0.93	2.00±1.26	-3.499 to 3.499
	Min 60	3.83±1.37	4.16±1.68	-5.175 to 4.508
	Min 120	5.00±1.06	3.83±0.91	-1.953 to 4.286
	Min 180	3.67±0.80	5.33±1.31	-5.086 to 1.753
	Min 240	3.83±0.65	5.50±1.15	-4.609 to 1.276
200 (mg/Kg)	Min 0	3.17±1.01	2.67±1.09	­2.809 to 3.809
	Min 30	1.50±0.50	2.33±0.61	­2.598 to 0.932
	Min 60	1.83±0.74	3.17±1.10	­4.313 to 1.646
	Min 120	2.83±1.22	3.33±0.88	­3.858 to 2.858
	Min 180	2.00±0.86	3.33±1.58	­5.346 to 2.680
	Min 240	3.17±1.19	2.83±1.17	­3.387 to 4.054
400 (mg/Kg)	Min 0	3.67±1.02	2.83±0.65	­1.870 to 3.536
	Min 30	0.83±0.30	1.00±0.52	­1.505 to 1.172
	Min 60	1.50±0.34	2.00±1.03	­3.153 to 2.153
	Min 120	2.33±0.92	2.00±0.82	-4.405 to 1.072
	Min 180	1.50±0.76	2.17±0.54	­2.754 to 1.420
	Min 240	3.50±0.72	3.83±1.17	­3.386 to 2.719

N.B: NS= Not Significant

**Table 7:** The effect of SKC (100, 200 and 400 mg/kg) in the total ambulation of hole board test.

Conc.	Group	Ctrl (n=6)	SKC (n=6)	95% confidence interval
100 (mg/Kg)	Min 0	19.00±4.02	20.83±4.88	-15.930 to 12.263
	Min 30	34.67±4.65	20.50±6.15	-3.038 to 31.372
	Min 60	39.67±8.08	15.67±4.20**	3.697 to 4.302
	Min 120	21.67±3.32	27.83±3.94	17.659 to 5.326
	Min 180	10.67±3.74	14.50±2.40	-13.738 to 6.071
	Min 240	10.83±3.55	14.83±3.84	-15.660 to 7.660
200 (mg/Kg)	Min 0	28.50±8.41	24.00±2.46	-17.073 to 26.073
	Min 30	31.33±12.81	13.00±5.91	-13.117 to 49.784
	Min 60	36.83±11.76	11.17±4.19**	-2.143 to 53.477
	Min 120	29.17±9.66	16.17±2.09	-9.033 to 35.033
	Min 180	31.33±8.89	20.33±4.05	-10.765 to 32.765
	Min 240	28.83±8.44	16.17±4.39	-8.548 to 33.881
400 (mg/Kg)	Min 0	23.17±7.03	18.00±3.18	-12.038 to 22.372
	Min 30	31.17±7.14	12.50±3.20**	1.234 to 36.099
	Min 60	18.83±6.95	14.67±3.64	-13.321 to 21.655
	Min 120	19.33±5.52	10.00±2.82	-4.493 to 23.160
	Min 180	21.83±2.68	14.67±3.23	-2.182 to 16.515
	Min 240	14.50±4.86	13.83±2.15	-11.181 to 12.515

** N.B: shows p<0.01 (highly significant).

**Table 8:** The effect of SKC (100, 200 and 400 mg/kg) in the head dipping of hole board test.

Conc.	Group	Ctrl (n=6)	SKC (n=6)	95% confidence interval
100 (mg/Kg)	Min 0	1.00±0.63	1.00±0.45	-1.725 to 1.725
	Min 30	0.67±0.42	1.83±1.32	-4.269 to 1.935
	Min 60	4.67±1.83	1.50±0.96	-1.664 to 7.998
	Min 120	2.33±0.56	2.17±1.22	-2.827 to 3.160
	Min 180	2.17±1.08	1.17±0.31	-1.496 to 3.496
	Min 240	0.50±0.34	0.00±0.00	-0.378 to 1.378
200 (mg/Kg)	Min 0	1.00±0.52	1.83±1.11	-3.557 to 1.890
	Min 30	2.50±1.18	0.17±0.17	-0.685 to 5.351
	Min 60	2.00±1.09	0.17±0.17	-0.977 to 4.644
	Min 120	2.33±1.23	0.00±0.00	-0.826 to 5.493
	Min 180	1.67±0.99	0.00±0.00	-0.875 to 4.208
	Min 240	1.00±1.00	0.00±0.00	-1.570 to 3.570
400 (mg/Kg)	Min 0	0.50±0.22	0.83±0.54	-1.836 to 1.069
	Min 30	0.33±0.33	0.17±0.17	-0.663 to 0.997
	Min 60	2.83±1.56	0.00±0.00	-1.171 to 6.838
	Min 120	0.67±0.49	0.17±0.17	-0.662 to 1.662
	Min 180	0.67±0.67	0.00±0.00	-1.047 to 2.380
	Min 240	0.67±0.49	0.17±0.17	-0.662 to 1.662

N.B: NS= Not Significant.

**Table 9.** The effect of SKC (100, 200 and 400 mg/kg) on the emotional defecation of hole board test

Conc.	Group	Ctrl (n=6)	SKC (n=6)	95% confidence interval
100 (mg/Kg)	Min 0	0.17±0.17	0.50±0.50	-1.507 to 0.841
	Min 30	0.33±0.33	1.00±0.52	-2.036 to 0.702
	Min 60	0.00±0.00	0.50±0.34	-1.378 to 0.378
	Min 120	0.00±0.00	0.67±0.33	-1.523 to 0.190
	Min 180	0.33±0.33	0.33±0.33	-1.050 to 1.050
	Min 240	0.33±0.33	0.67±0.67	-0.663 to 0.997
200 (mg/Kg)	Min 0	0.67±0.42	1.50±0.62	-2.502 to 0.835
	Min 30	1.33±0.61	0.17±0.17	-0.252 to 2.585
	Min 60	1.67±0.21	0.50±0.34	0.272 to 2.061
	Min 120	2.00±0.45	0.83±0.31	-0.042 to 2.375
	Min 180	2.17±0.31	1.17±0.40	-0.126 to 2.126
	Min 240	2.50±0.56	1.00±0.52	-0.201 to 3.021
400 (mg/Kg)	Min 0	2.67±0.84	2.83±0.60	-2.473 to 1.400
	Min 30	2.67±0.56	2.17±0.48	-1.135 to 2.135
	Min 60	1.33±0.61	2.00±0.52	-2.455 to 1.122
	Min 120	2.17±1.01	2.00±0.86	-2.790 to 3.123
	Min 180	2.17±0.31	2.83±0.65	-2.276 to 0.943
	Min 240	2.33±0.80	2.00±0.86	-2.282 to 2.948

N.B: NS= Not Significant.

**Figure 1:**
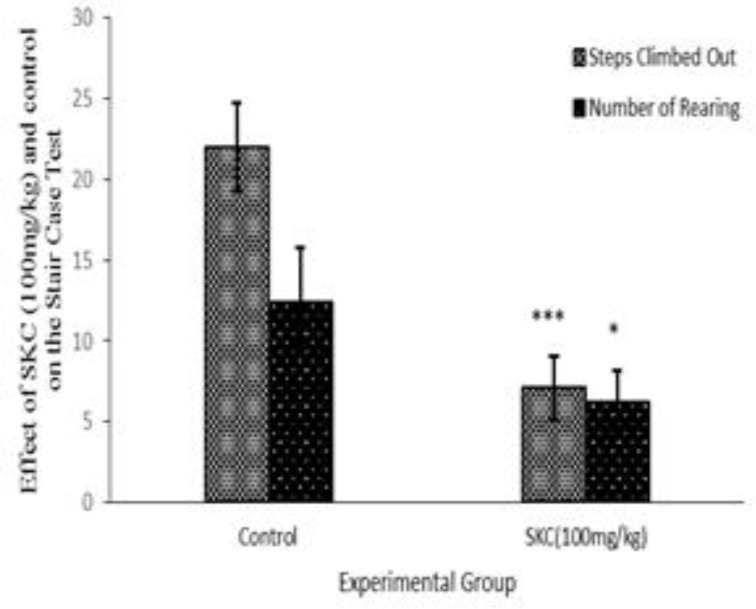
. Tabular presentation of the effect of SKC (100 mg/kg) on the Stair Case Test utilizing male mice. F.N :* indicates P<0.05 (Significant), ** indicates P<0.01 (Highly Significant) and *** indicates P<0.001(Very Highly Significant

SKC 100mg/kg (male mice) group decreased both the locomotor (p<0.001) and the number of rearing (p<0.01) in the staircase test, as compared to the corresponding control group. It can be suggested that SKC has mild anxiolytic activity (Figure 1).

**Figure 2:**
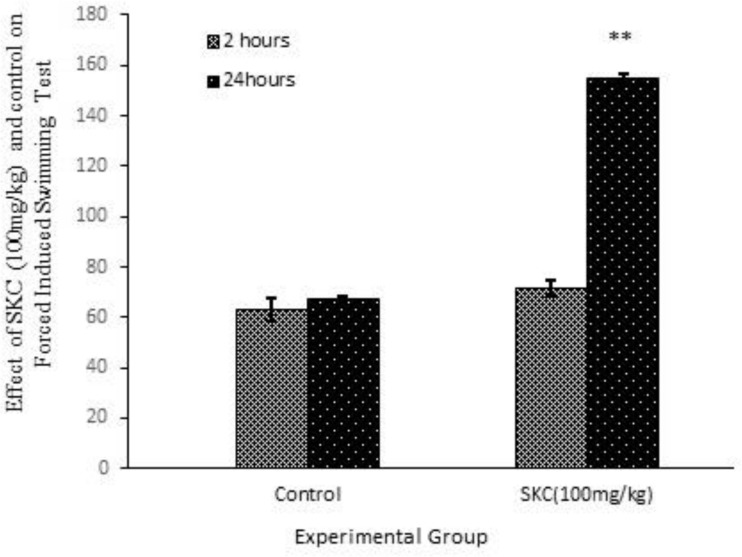
Graphical presentation of the effect of SKC (100 mg/kg) on the Forced Induced Swimming Test utilizing male mice.

In the force induced swimming test, experimental group treated with SKC 100mg/kg showed an overall increase in immobile phase compared to their corresponding control group (Figure. 2). The rate of increasing immobile phase was much greater at the 24^th^ hr than 2^nd^ hr after drug administration. At the end of 24^th^ hr, we found an increase in immobile phase which was statistically highly significant (p<0.01). In the rota rod test, male mice treated with SKC 100, 200 and 400 mg/kg revealed an overall decrease in total fall and data obtained was comparable to the respective control group with an exception at the dose level of 100mg/kg at min 240 (Table 12).

**Table 10:** The effect of SKC (100, 200 and 400 mg/kg) on the time spent in open arms.

Conc.	Group	Ctrl (n=6)	SKC (n=6)	95% confidence interval
100 (mg/Kg)	Min 0	2.67±2.29	0.50±0.50	­3.056 to 7.389
	Min 60	9.00±4.27	4.00±2.58	­6.125 to 16.125
	Min 120	3.67±2.65	0.50±0.50	­3.640 to 9.973
	Min 180	1.00±0.68	1.17±0.74	­2.425 to 2.092
	Min 240	5.17±4.25	0.83±0.54	­5.204 to 13.870
200 (mg/Kg)	Min 0	0.00±0.00	0.00±0.00	0.000 to 0.000
	Min 60	2.00±2.00	0.00±0.00	­3.141 to 7.141
	Min 120	0.50±0.50	1.00±1.00	­2.991 to 1.991
	Min 180	0.67±0.67	0.00±0.00	­1.047 to 2.380
	Min 240	3.00±2.16	2.00±2.00	­5.559 to 7.559
400 (mg/Kg)	Min 0	0.83±0.83	0.50±0.50	­1.832 to 2.498
	Min 60	0.00±0.00	0.00±0.00	0.00 to 0.00
	Min 120	2.00±0.93	0.00±0.00	.393 to 4.393
	Min 180	4.17±3.60	0.83±0.83	­4.900 to 11.567
	Min 240	6.50±3.36	0.00±0.00	­2.147 to 15.147

N.B: NS= Not significant.

**Table 11:** The effect of SKC (100, 200 and 400 mg/kg) in the total movement in close arms.

Conc.	Group	Ctrl (n=6)	SKC (n=6)	95% confidence interval
100 (mg/Kg)	Min 0	10.50±1.43	10.50±2.86	­7.127 to 7.127
	Min 60	7.50±2.46	5.00±1.59	­4.027 to 9.027
	Min 120	4.83±1.68	2.83±1.10	­2.487 to 6.487
	Min 180	3.00±0.89	4.16±1.72	­5.689 to 3.355
	Min 240	5.67±1.47	8.17±2.55	­9.061 to 4.061
200 (mg/Kg)	Min 0	4.17±1.30	4.83±1.83	­5.676 to 4.343
	Min 60	6.67±1.40	3.83±1.22	­1.318 to 6.985
	Min 120	6.83±2.31	2.83±1.22	­1.833 to 9.833
	Min 180	6.00±1.15	5.67±0.84	­2.852 to 3.519
	Min 240	6.83±0.703	6.00±1.77	­3.735 to 5.402
400 (mg/Kg)	Min 0	6.17±1.14	4.33±1.89	­3.084 to 6.571
	Min 60	2.67±0.76	1.83±0.31	­1.129 to 2.796
	Min 120	4.50±.1.65	3.00±0.93	­2.717 to 5.717
	Min 180	5.00±.2.17	2.67±.1.26	­3.264 to 7.930
	Min 240	4.00±.1.24	2.67±.1.28	­2.638 to 5.305

N.B: NS= Not Significant.

N.B: ** shows P<0.01 (Highly Significant

**Table 12:** The effect of SKC (100, 200 and 400 mg/kg) on the total fall in the rota rod test

Conc.	Group	Ctrl (n=8)	SKC (n=8)	95% confidence interval
100 (mg/Kg)	Min 0	1.75±0.59	1.50±0.65	-1.640 to 2.140
	Min 30	1.87±0.69	1.87±0.93	-2.494 to 2.494
	Min 60	0.87±0.29	0.50±0.27	-0.478 to 1.228
	Min 120	0.25±0.16	0.75±0.31	-1.282 to 0.282
	Min 180	0.62±0.26	0.62±0.42	-1.062 to 1.062
	Min 240	0.62±0.26	0.50±0.19	-0.569 to 0.189
200 (mg/Kg)	Min 0	9.25±3.11	4.50±2.07	-3.272 to 12.772
	Min 30	6.75±1.84	3.13±1.79	-1.891 to 9.141
	Min 60	4.75±1.80	2.00±0.91	-1.572 to 7.072
	Min 120	5.25±2.17	2.63±2.07	-3.831 to 9.081
	Min 180	4.00±2.00	3.00±2.29	-5.535 to 7.535
	Min 240	4.13±1.87	3.37±2.69	-6.286 to 7.786
400 (mg/Kg)	Min 0	6.00±1.66	2.75±0.84	-0.871 to 7.371
	Min 30	5.50±1.40	5.37±1.61	-4.459 to 4.709
	Min 60	6.50±2.57	3.13±1.63	-3.153 to 9.903
	Min 120	6.12±1.71	4.00±2.87	-5.05 to 9.31
	Min 180	4.37±1.73	3.75±2.77	-6.391 to 7.641
	Min 240	4.25±1.56	5.25±2.95	-8.165 to 6.165

N.B: NS= Not Significant.

## Discussion

In the present study, Swas Kas Chintamani Ras (SKC) was evaluated for the psychopharmacological and neurosafety properties. Despite intensive efforts made to discover novel psychiatric drugs for psychotic and anxiety disorders over the past two decades, unfortunately, all drugs have shown marked side effects. In this respect, Ayurvedic medications could be an attractive candidate as therapeutic strategies for treatment of these conditions (Calixto et al., 2000 ▶; Fisher et al., 1994 ▶). Reduction in the locomotor activity indicates CNS depressant property of a drug. SKC increased loco motor activity at 4th hr at 100 mg/kg but when doses increased, this effect was only observed at the very beginning of the experimental period. All doses of SKC increased interest in crossing the hole but decreased ambulation and head dipping activity which supporting neuro safety status of this drug. Concerning psychopharmacological effects of the drug, mice spent less time to come out of the cage, but only at min 60, they spent much longer duration than any other examined dose. They also spent shorter time in open arm and had less movement in the closed arm and locomotors, where the number of rearing behavior indicating possible anxiety or anxiolytic activity. EPM test is one of the most frequently used animal models in behavioral psychopharmacology for screening drugs with potential anxiolytic effects (Wall et al., 2000 ▶). In general, reduction or increase in the number of entries and times spent in the open arms induced by a given substance, had been regarded as good indicators of its anxiogenic or anxiolytic effects, respectively (Pellow et al., 1985 ▶). The present findings reveal that administration of SKC could exhibit the anxiolytic-like effect in this paradigm. This may be due to modulation of GABA receptors by SKC. During this study, SKC did not produce any considerable changes in the elevated plus maze model. The exposure of mice to an elevated and open maze induces an exploratory cum fear drive which results in anxiety (Handley and McBlane et al., 1993 ▶; Kannan et al., 2011 ▶). Anxiolytic substances are act by ameliorating the open arm exploration, decreasing anxiety, as well as increasing the number of entries into the open arm. The SKC successfully showed such potentials at both doses demonstrating that the plant at the studied doses possesses anxiolytic activity. The result of stair case test revealed that treatment with SKC significantly decreases locomotor activities and the number of rearing behavior indicating possible anxiolytic activity. No anti-depressant activity was observed in forced swimming test among SKC-treated group. When mice are forced to swim in an inescapable situation, they tend to become immobile after initial vigorous activity. The immobility reflects a state of lowered mood in which the animals give up hope of finding an exit and resign to the experimental situation. This absence of immobility has been described as a symptom of “behavioral despair” (Karolewiaz et al., 2001 ▶). Also, SKC showed significant decreases in immobility time enforcing antidepressant activity of the plant extract, which is opposite to the effect shown by classical antidepressant drugs like fluoxetine (Porsolt, 1977; Willner et al., 1990 ▶). Several previous studies have supported the present findings (Borsini et al., 1988 ▶; Yonko et al., 1984 ▶; Novas et al., 1988 ▶). In the present, we aimed to examine whether metals present in the formulation induce any neurological or psychological toxicity or not. But fortunately, not only we found no neurotoxicity following administration of this Ayurveda formulation, but also we found some medicinal activities.

Our results confirmed that SKC possesses significant antidepressant, antipsychotic and anxiolytic activity. The results are encouraging to pursue further studies to discover the underlying mechanisms and also to isolate and characterize responsible bioactive molecule(s). The result of this present study may be create better pathway to generate preparation for drugs with neuro safety and alternative pathway to reveal other medical value of any established herbal preparation. Thus, further research must be devoted towards determination of the qualitative and quantitative composition of SKC and isolation of biologically active compounds with full elucidation of precise mechanisms of activity. With regard to the currently available medical treatments for psychiatric disorders, the results obtained from the present research seem to be important because not only anxiolytic effects were observed but also antidepressant activity was also shown by SKC. These behavioral effects were supported by some other previous findings treating mice with classical antidepressant drugs. Moreover, SKC did not modify the spontaneous locomotor activity of the animals, therefore, it is probable that these effects are not mediated by stimulation of general motor activity. 
